# House Governor or Medical Superintendent?—III

**Published:** 1892-08-27

**Authors:** 


### PASSING TOPICS.

### HOUSE GOVERNOR OR MEDICAL SUPERINTENDENT?—III. (*Conclusion.*)

If the chief executive officer of a hospital could be made to combine in his own person and character the recommendations of a good business man, freedom from professional prejudices, and the advantages of a medical training, no doubt the outlook would be promising. But the combination of the best qualities of a doctor and a layman is practically an impossibility. They will seldom blend.

Only those who have an intimate knowledge of hospital life can form a true conception of the impatience of lay influence and control, exhibited by most medical officers. Not even the receipt of a salary suffices to ensure respect for lay authorities. The paid medical officers of a hospital take their stipends from the committee, but their services are rendered to the staff. A member of the profession is medical first whatever else he may be afterwards. He is subject to rules, written and unwritten, as rigorous as can be devised, and in his relation to the profession his individual liberties are small. It follows that a medical superintendent, even though he received his appointment independently of the medical and surgical staff, and this could scarcely happen, would yet come under their control immediately he entered upon his duties.

No matter how liberal minded he might by nature be, or how free from those prejudices which are inimical to the "manysidedness" of hospital work, he could not be relied upon to take a course independent of the staff, whose devoted servant he must soon become by force of circumstances. One result would be a gulf between the governing body and the affairs governed, and instead of being in touch, as they ought to be with all that is comprehended in the complex life of the institution, the so-called managers would depend for information and for guidance upon an official hedged about with technicalities, and actually, if not openly opposed to their interference.

It may be asked why lay and medical authority must necessarily conflict? The answer is because the object in view is not quite the same in both cases. We have no intention of depicting our hospitals as arenas of never-ending strife, but keen diversity of view is not less there because mutual good sense and forbearance prevent the frequent occurrence of unseemly disputes. This diversity—which may not be wholly disadvantageous—shows itself in many different ways, and while hospitals are regarded by the one party as institutions maintained primarily in the interests of general medicine and surgery, and by the other as places for affording relief to individual sickness and suffering, no further reason for it need be sought.

To ensure the confidence of the subscribing classes, the maintenance of the lay element in hospital control is indispensable. The exigencies of hospital finance therefore supply an unanswerable argument against undiluted medical management, and beyond that we have to remember that the confidence of the sick poor is also an important consideration. At present, notwithstanding the efforts made in certain quarters to represent the contrary, the hospitals enjoy that in full measure. But the same causes which would lead to a secession of subscribers would operate unfavourably in respect of the patients. Their confidence is derived from a well-founded belief in the philanthropic character of hospital work, and it extends, as it ought, to the medical officers. But it would be folly to suppose that either patients or subscribers are not fully alive to the difference between hospitals as they are, and hospitals made "medical throughout." Lay government, if it survived at all, would approximate to a sham whenever it was forced to depend upon a medical executive. AGRICOLA.

